# Integrating Cardiopulmonary Exercise Testing, Stress Echocardiography and Near-Infrared Spectroscopy for Multimodal Assessment of Exercise Intolerance: A Narrative Review

**DOI:** 10.3390/healthcare14111511

**Published:** 2026-05-29

**Authors:** Geza Halasz, Raffaella Mistrulli, Marco Di Francesco, Guido Giacalone, Gianluca Ferri, Stefano Beato, Francesca Moschella Orsini, Giovanni Nardecchia, Bernadette Corica, Furio Colivicchi, Stefania Angela Di Fusco, Federica Re, Domenico Gabrielli

**Affiliations:** 1 Division of Cardiology, Cardio-Thoracic and Vascular Department, Azienda Ospedaliera San Camillo Forlanini, Cir.ne Gianicolense 87, 00152 Rome, Italy; 2 Department of Clinical and Molecular Medicine, Sapienza University of Rome, 00185 Rome, Italy; 3 Department of Cardiovascular Sciences, Fondazione Policlinico Universitario Campus Bio-Medico, 00128 Rome, Italy; 4 Cardiology Unit, National Institute for Infectious Diseases Lazzaro Spallanzani, 00149 Rome, Italy; fmoschellaorsini@gmail.com; 5 Faculty of Medicine and Surgery, Sapienza University of Rome, 00185 Rome, Italy; 6 Cardiology Division, Department of Biomedical, Metabolic and Neural Sciences, University of Modena and Reggio Emilia, Policlinico di Modena, 41125 Modena, Italy; 7 Clinical and Rehabilitation Cardiology Unit, San Filippo Neri Hospital, ASL Roma 1, 00135 Rome, Italy

**Keywords:** cardiopulmonary exercise testing, stress echocardiography, near-infrared spectroscopy, exercise intolerance, skeletal muscle oxygenation, heart failure, pulmonary hypertension, cardiomyopathies, sports cardiology

## Abstract

Cardiopulmonary exercise testing (CPET) is the reference method for the objective assessment of exercise capacity because it provides an integrated appraisal of cardiovascular, respiratory and metabolic responses to exertion. However, CPET alone quantifies the magnitude of functional impairment without fully resolving the central and peripheral mechanisms that determine exercise intolerance. The integration of CPET with exercise stress echocardiography and near-infrared spectroscopy (NIRS) has therefore emerged as a clinically relevant multimodal strategy. Stress echocardiography provides real-time information on ventricular reserve, filling pressures, pulmonary pressure response, valvular function, pulmonary congestion and dynamic outflow obstruction, whereas NIRS provides continuous insight into skeletal muscle oxygen delivery, extraction and utilization. This narrative review summarizes the physiological rationale, practical workflow, methodological limitations and clinical applications of combined CPET, stress echocardiography and NIRS across heart failure, pulmonary hypertension, peripheral artery disease, cardiomyopathies and sports cardiology. By linking systemic gas exchange, central hemodynamics and peripheral oxygen handling, this approach may move exercise evaluation from a descriptive measure of performance toward a mechanism-based framework for phenotyping, risk stratification and individualized therapeutic decision-making. Further studies are needed to harmonize protocols, validate reproducible multimodal indices and demonstrate incremental prognostic value over conventional testing.

## 1. Introduction

Over the last decades, the evaluation of exercise intolerance has progressively evolved from a predominantly descriptive approach to a more integrated and mechanistic interpretation of functional limitation. In this context, cardiopulmonary exercise testing (CPET) has become the reference method for the objective assessment of exercise capacity, providing a global and dynamic evaluation of the cardiovascular, respiratory, and metabolic responses to effort. By measuring variables such as oxygen uptake (VO_2_), carbon dioxide production (VCO_2_), minute ventilation (VE), ventilatory efficiency, and anaerobic thresholds, CPET allows clinicians to quantify functional capacity and identify the principal determinants of exercise limitation. Its major strength lies in the ability to move beyond symptoms alone and provide a reproducible physiological profile of exercise performance. However, CPET does not directly distinguish the relative contribution of central hemodynamic abnormalities and peripheral muscular dysfunction to exercise limitation. Peak VO_2_, VE/VCO_2_ slope, and other derived variables remain the final expression of complex interactions among cardiac output, pulmonary function, peripheral oxygen delivery, and skeletal muscle utilization.

To overcome these limitations, the combination of CPET with stress echocardiography has emerged as an important step forward in the functional assessment of patients with cardiovascular disease. This integrated approach enables the simultaneous evaluation of metabolic exercise responses and real-time cardiac structure and function during stress. By coupling gas exchange analysis with exercise-induced changes in left ventricular filling pressures, pulmonary pressures, contractile reserve, stroke volume, and dynamic outflow or valvular abnormalities, CPET-stress echocardiography provides a more comprehensive interpretation of exercise intolerance. Rather than simply documenting reduced exercise capacity, it allows clinicians to identify the central hemodynamic substrate underlying performance impairment and to place functional abnormalities into a pathophysiological context [1,2].

More recently, near-infrared spectroscopy (NIRS) has added a further dimension to exercise evaluation by extending the analysis from the central circulation to the peripheral tissues. NIRS is a non-invasive optical technique that continuously monitors local muscle oxygenation through the differential absorption of near-infrared light by oxygenated and deoxygenated hemoglobin. Applied during exercise, it provides real-time information on regional oxygen delivery, extraction, and utilization within skeletal muscle, thereby offering insight into the peripheral component of exercise performance. This is particularly relevant because exercise intolerance is often not exclusively cardiac in origin, but may also reflect abnormalities in microvascular function, muscle perfusion, oxidative metabolism, or mitochondrial capacity [3,4].

In this framework, each modality provides a distinct and complementary perspective: CPET quantifies systemic gas exchange and overall functional capacity, stress echocardiography characterizes central hemodynamic reserve and dynamic cardiac abnormalities during exertion, whereas NIRS explores local peripheral oxygenation and skeletal muscle microvascular–metabolic behavior.

The integration of CPET, stress echocardiography, and NIRS therefore represents an important evolution in exercise physiology and clinical cardiology. This multimodal approach enables a multidimensional interpretation of exercise intolerance by integrating systemic performance, central hemodynamics, and peripheral oxygen handling. Such a comprehensive strategy may improve phenotyping, refine diagnostic accuracy, and better identify the dominant mechanisms responsible for functional limitation across a broad spectrum of cardiovascular and systemic conditions. In this evolving framework, exercise testing is no longer merely a tool for measuring functional capacity, but also an instrument for understanding the biological mechanisms underlying impaired performance.

Despite the growing interest in multimodal assessment of exercise intolerance, the combined use of cardiopulmonary exercise testing, stress echocardiography, and near-infrared spectroscopy remains insufficiently standardized and is often applied in a fragmented manner across clinical and research settings. Current literature predominantly focuses on single-modality approaches or, at most, dual combinations, while a comprehensive integration of systemic, central, and peripheral components of exercise physiology is still lacking.

In this context, the aim of the present narrative review is to provide a clinically oriented synthesis of the physiological rationale, methodological considerations, and potential applications of an integrated CPET–stress echocardiography–NIRS approach. In addition, we seek to highlight current limitations, identify gaps in standardization and interpretation, and propose a practical framework for the implementation of multimodal exercise assessment in different clinical scenarios.

## 2. Methods

This article was designed as a narrative review aimed at providing a clinically oriented synthesis of the physiological rationale, methodology, and potential applications of integrated cardiopulmonary exercise testing (CPET), exercise stress echocardiography, and near-infrared spectroscopy (NIRS). A targeted literature search was conducted using major electronic databases, including PubMed/MEDLINE and Scopus, focusing on publications related to CPET, stress echocardiography, exercise hemodynamics, skeletal muscle oxygenation, and multimodal exercise assessment. Relevant literature was identified using combinations of thematic keywords related to cardiopulmonary exercise testing, stress echocardiography, near-infrared spectroscopy, heart failure, pulmonary hypertension, peripheral artery disease, cardiomyopathies, and sports cardiology. Priority was given to consensus documents, guideline statements, methodological papers, clinical studies, and recent clinically relevant evidence. Articles were selected based on scientific relevance, methodological quality, and their contribution to the understanding of integrated multimodal exercise evaluation. The review does not report new patient-level data and was intended to critically organize available evidence, highlight current limitations, and discuss potential practical applications and future research directions. Although no formal systematic review or standardized narrative review reporting framework was applied, the manuscript was developed using a structured narrative approach aimed at critically synthesizing clinically relevant and methodologically robust evidence across the main domains of multimodal exercise assessment. This should be considered a limitation of the present review.

## 3. Core Modalities: CPET, Stress Echocardiography and Near-Infrared Spectroscopy

### 3.1. Cardiopulmonary Exercise Testing

Cardiopulmonary exercise testing (CPET) is the reference methodology for the integrated assessment of exercise performance, as it simultaneously evaluates the respiratory, cardiovascular, and metabolic responses to progressive physical stress. It is typically performed using an incremental ramp protocol on a cycle ergometer or treadmill, with breath-by-breath analysis of expired gases. CPET directly measures oxygen uptake (VO_2_), carbon dioxide production (VCO_2_), and minute ventilation (VE), from which several clinically relevant parameters are derived, including peak VO_2_, ventilatory thresholds, VE/VCO_2_ slope, oxygen pulse, respiratory exchange ratio, and breathing reserve. These variables provide an objective quantification of functional capacity and help identify the mechanisms responsible for exercise limitation. Because CPET reflects the integrated performance of multiple organ systems under stress, it is particularly useful in distinguishing whether exercise intolerance is mainly related to circulatory impairment, ventilatory abnormalities, peripheral deconditioning, or altered oxygen utilization. However, although CPET provides a robust systemic overview, it does not directly visualize cardiac mechanics or regional peripheral oxygen dynamics [5,6].

### 3.2. Stress Echocardiography

Stress echocardiography is a dynamic imaging modality that assesses cardiac structure and function during exercise or pharmacological stress. In the context of exercise physiology, semi-supine or upright exercise echocardiography is especially valuable because it allows real-time evaluation of hemodynamic and functional changes under conditions that reproduce symptoms. Compared with resting echocardiography, stress echocardiography can detect abnormalities that only emerge during exertion, such as impaired contractile reserve, elevated left ventricular filling pressures, exercise-induced pulmonary hypertension, worsening mitral regurgitation, dynamic left ventricular outflow tract obstruction, or regional wall motion abnormalities suggestive of myocardial ischemia. It also enables the assessment of stroke volume reserve, right ventricular performance, and the behavior of pulmonary pressures during exercise. Therefore, stress echocardiography complements functional data by providing direct insight into the central cardiac and hemodynamic determinants of effort intolerance. Its main strength lies in the ability to link symptoms and reduced performance to a specific pathophysiological cardiac substrate [7,8].

### 3.3. Near-Infrared Spectroscopy

Near-infrared spectroscopy (NIRS) is a non-invasive optical technique used to monitor local tissue oxygenation continuously and in real time, particularly at the level of skeletal muscle and, in selected applications, cerebral tissue. NIRS is based on the differential absorption of near-infrared light, usually within the 700–1000 nm spectrum, by oxygenated and deoxygenated hemoglobin. After light penetrates biological tissues, the detected changes in absorbance are analyzed using the modified Beer-Lambert law to estimate relative changes in oxygenated hemoglobin, deoxygenated hemoglobin, total hemoglobin, and tissue saturation index. When applied during exercise, NIRS provides regional information on oxygen delivery, extraction, and utilization within the interrogated muscle, thereby offering a unique window into the peripheral component of exercise performance. In addition, when combined with arterial occlusion protocols, NIRS recovery kinetics may serve as an indirect marker of skeletal muscle oxidative capacity. Unlike CPET, which captures whole-body responses, and stress echocardiography, which focuses on central hemodynamics, NIRS specifically explores peripheral muscle oxygen handling and microvascular–metabolic behavior [3,9,10]. However, despite its potential, this technology presents several technical and methodological challenges. First, signal quality can be influenced by factors such as skin pigmentation, adipose tissue thickness, and probe positioning, which can affect the accuracy and reproducibility of measurements. Inter-subject variability is often high, requiring standardized protocols and normalization procedures to ensure meaningful comparisons. Additionally, common NIRS devices used in clinical practice (e.g., continuous-wave NIR spectrometers) provide relative rather than absolute oxygenation values, which limits their standalone diagnostic utility. Although the Tissue Saturation Index (TSI) is designed to overcome this issue, its underlying algorithms are strictly device-dependent and may vary significantly across different NIRS platforms. The clinical interpretation of NIRS-derived variables, particularly deoxyhemoglobin (HHb) and the TSI, remains one of the most significant challenges for the widespread adoption of this technology. While HHb is widely regarded as a proxy for fractional tissue oxygen extraction—reflecting the balance between local O_2_ delivery and utilization—its absolute quantification is often hindered by inter-subject variability. A primary critical issue is the current lack of definitive, universally accepted “cut-off” values for clinical diagnosis. Unlike parameters derived from CPET (such as peak VO_2_ or the VE/VCO_2_ slope), NIRS variables suffer from a lack of large-scale standardized normative data, making it difficult to distinguish between a “normal” and a “pathological” muscle oxygenation dynamic based solely on absolute numbers. Furthermore, interpretation must be approached with caution, as this percentage reflects the relative saturation of the small vessel heme group within the illuminated tissue volume. Factors such as skin microcirculation, subcutaneous fat thickness, and the heterogeneity of fiber recruitment can confound the signal. Consequently, rather than relying on static thresholds, clinicians should focus on the kinetics of these variables—such as the slope of deoxygenation during incremental exercise or the speed of reoxygenation during recovery—which may offer more robust clinical insights.

## 4. Rationale and Methodology for Integrated CPET, Stress Echocardiography and NIRS

### 4.1. Complementary Physiological Domains

The integration of cardiopulmonary exercise testing (CPET), stress echocardiography (ESE), and near-infrared spectroscopy (NIRS) enables a comprehensive assessment of exercise intolerance by simultaneously exploring systemic performance, central hemodynamics, and peripheral oxygen handling. Although each modality yields relevant information when used alone, their combined application allows a more precise identification of the mechanisms limiting functional capacity. CPET remains the cornerstone of exercise evaluation because it quantifies the overall physiological response to exertion through gas exchange, ventilatory efficiency, and metabolic adaptation. Peak oxygen uptake (VO_2_peak), the most widely adopted measure of aerobic performance, is determined by the interaction between cardiac output and peripheral oxygen extraction according to the Fick principle.

However, standard CPET does not directly resolve the relative contribution of central versus peripheral factors to exercise limitation. Stress echocardiography provides this missing hemodynamic context. Assessing cardiac structure and function during exercise allows for a direct evaluation of biventricular reserve, stroke volume adaptation, exercise-induced changes in filling pressures, pulmonary pressures, valvular function, and dynamic intraventricular obstruction. In this way, abnormal CPET responses can be interpreted within a structural and functional framework, clarifying whether reduced exercise capacity is primarily related to impaired forward flow, abnormal diastolic reserve, pulmonary vascular burden, or exertional congestion [11].

NIRS adds a complementary peripheral perspective by continuously monitoring local muscle oxygenation during exercise. Through the analysis of oxygenated and deoxygenated hemoglobin signals, it provides indirect information on regional oxygen delivery, extraction, and utilization. This offers a unique opportunity to investigate the peripheral component of the arteriovenous oxygen difference, which is not directly captured by conventional CPET. Such information may be particularly valuable in conditions characterized by skeletal muscle hypoperfusion, microvascular dysfunction, impaired oxidative metabolism, or mitochondrial abnormalities. Taken together, CPET, ESE, and NIRS interrogate distinct but interdependent domains of exercise physiology. Their integration therefore supports a more refined pathophysiological interpretation of exercise intolerance and may improve phenotyping, risk stratification, and therapeutic personalization [12]. From a clinical perspective, this integrated approach may help orient therapeutic decision-making by identifying whether exercise limitation is predominantly related to impaired central hemodynamics, pulmonary vascular dysfunction, or peripheral muscular abnormalities, potentially supporting more individualized pharmacological, rehabilitative, and follow-up strategies.

### 4.2. Proposed Integrated Protocols and Practical Workflow

Currently, no standardized or widely accepted protocols exist for the combined application of CPET, ESE, and NIRS [13]. However, based on currently available published evidence, physiological rationale, and expert-oriented interpretation of multimodal exercise assessment, certain practical recommendations may be proposed for clinical and research implementation: A potential integrated workflow may include the following components:

- Pre-test: Baseline spirometry is recommended to identify obstructive or restrictive patterns.
- Exercise protocol: A total exercise duration of 8–12 min with a progressive ramp protocol (8–20 W/min) is advised, in accordance with CPET guidelines [1]. Continuous ECG, blood pressure, peripheral oxygen saturation, and breath-by-breath gas exchange monitoring are essential. The exercise protocol must be performed following a brief warm-up phase for initial NIRS signal stabilization, system calibration, and adaptation to pedaling-related motion artifacts
- Echocardiographic imaging: To be acquired at rest, low workload (<100 bpm), anaerobic threshold, and peak exercise. Parameters to assess include left ventricular systolic and diastolic function, valvular abnormalities, cardiac output (calculated via LVOT VTI), right ventricular function (e.g., TAPSE, TAPSE/PASP), pulmonary arterial pressure (PASP), and pulmonary congestion (assessed via B-lines on lung ultrasound).
- NIRS monitoring: The sensor should be positioned over a target muscle (e.g., quadriceps femoris muscle), ensuring secure fixation to minimize motion artifacts and reduce ambient light interference. Subcutaneous adipose tissue thickness (ATT) should be considered due to its impact on signal quality. The monitoring protocol follows a structured timeline to ensure data reliability:
  - **Warm-up and Calibration:** A preliminary phase is essential for signal stabilization and baseline calibration. During the warm-up, the system adjusts to motion artifacts inherent to the pedaling action, ensuring that subsequent readings are physiologically grounded.
  - **Continuous Measurement:** Data acquisition must remain uninterrupted throughout the exercise phase and the subsequent recovery phase.
  - **Active Recovery and Termination:** To prevent abrupt changes in limb position from compromising the sampling accuracy, the patient should continue pedaling during the recovery phase. Monitoring should only be terminated once the variables have reached a post-exercise plateau (i.e., when parameters no longer show significant fluctuations).
  Key parameters include:
- O_2_Hb (Oxyhemoglobin): Represents the amount of hemoglobin that is bound to oxygen. A decrease during exercise typically suggests that the local metabolic demand is exceeding the oxygen supply being delivered by the blood flow.
- HHb (Deoxyhemoglobin): Represents hemoglobin that has released its oxygen (reduced hemoglobin). It is considered a highly sensitive indicator of tissue oxygen extraction.
- tHb (Total Hemoglobin): Sum of O_2_Hb and HHb, serves as a proxy for local blood volume in the tissue under the sensor
- DiffHb (Hemoglobin Difference): Difference between O_2_Hb and HHb, used as an index of tissue O_2_ extraction
- TSI (Tissue Saturation Index): The ratio between O_2_Hb and tHb (expressed as a percentage). Reflects an index of changes in tissue O_2_ saturation, indicating the balance between oxygen delivery and consumption.

**Figure 1:**
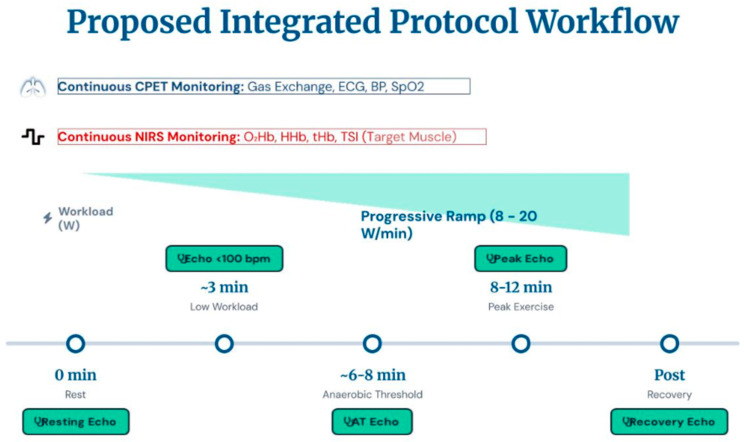
Proposed practical workflow for integrated cardiopulmonary exercise testing (CPET), exercise stress echocardiography (ESE), and near-infrared spectroscopy (NIRS) assessment. The figure illustrates the conceptual integration of systemic gas exchange analysis, central hemodynamic evaluation, and peripheral muscle oxygenation monitoring during exercise and recovery phases. Abbreviations: AT, anaerobic threshold; HHb, deoxyhemoglobin; LVOT VTI, left ventricular outflow tract velocity–time integral; O_2_Hb, oxyhemoglobin; PASP, pulmonary artery systolic pressure; TSI, tissue saturation index.

The integrated use of these tools, although technically demanding, provides a multidimensional view of exercise physiology (Figure 1). The interpretation of integrated CPET–stress echocardiography–NIRS findings should consider the potential dissociation between central hemodynamic impairment and peripheral muscular dysfunction. For example, reduced peak VO_2_ associated with abnormal filling pressures, impaired cardiac output reserve, or exercise-induced pulmonary hypertension may suggest a predominantly central limitation, whereas preserved central hemodynamics with impaired muscle oxygen extraction or delayed recovery kinetics on NIRS may support a greater peripheral contribution. Mixed phenotypes are likely common, particularly in chronic heart failure and deconditioned patients, highlighting the importance of integrated multimodal interpretation. At present, evidence supporting these integrated approaches mainly derives from observational cohorts, mechanistic studies, and pilot investigations rather than large prospective validation studies or standardized protocols.

### 4.3. Potential Artifacts and Limitations

Despite its diagnostic potential, the combined CPET-ESE-NIRS approach presents several limitations. First, there is a lack of standardization: there are currently no official guidelines or shared protocols, leading to inter-center variability. Furthermore, there is a need for dedicated infrastructure: access to integrated equipment (cycle ergometer, gas analyzer, echocardiograph, NIRS device) and appropriate testing space may be limited. Technical challenges are also prominent: echocardiographic acquisition during exercise is susceptible to motion and respiratory artifacts and requires substantial operator expertise. Crucially, the complexity of this integrated assessment makes it difficult to perform by a single operator, necessitating a multidisciplinary team of several people to synchronize data collection. NIRS is likewise vulnerable to motion artifacts, probe displacement, ambient light interference, and attenuation of the optical signal by adipose tissue. To mitigate these issues, the protocol must include a preliminary warm-up phase for signal stabilization and calibration, allowing the system to adapt to pedaling-related artifacts. Measurement must then remain continuous through both exercise and recovery phases; notably, the patient should continue pedaling during recovery so that motion artifacts remain consistent and do not bias the sampling. Monitoring should only be interrupted once the variables have reached a stable post-exercise plateau. In addition, interpretation of NIRS-derived variables remains partly device-specific and requires dedicated expertise, although NIRS monitoring and data acquisition can be effectively managed by appropriately trained non-medical personnel.

Finally, patient limitations must be considered: not all individuals can perform maximal exercise tests (e.g., due to severe obesity, orthopedic issues, or hemodynamic instability). Despite these barriers, the integration of CPET, ESE, and NIRS represents a promising frontier for personalized functional assessment, with potential applications in both clinical practice and research settings [13].

## 5. Results and Discussion: Clinical Applications of Multimodal Exercise Assessment

Functional assessment in cardiology relies on advanced methods that enable an integrated analysis of hemodynamic, ventilatory, and metabolic responses to exercise. Among these, CPET, Stress echocardiography, and near-infrared spectroscopy (NIRS) are complementary diagnostic tools that play a crucial role in prognostic stratification, differential diagnosis, and therapeutic optimization in patients with cardiovascular diseases.

### 5.1. Heart Failure

Heart failure (HF) is a complex clinical syndrome characterized by marked pathophysiological heterogeneity and substantial prognostic burden. Because exercise intolerance in HF may result from different and often overlapping mechanisms, including impaired cardiac output, abnormal filling pressures, pulmonary vascular dysfunction, ventilatory inefficiency, and peripheral skeletal muscle abnormalities, an integrated functional assessment is particularly valuable for a more comprehensive characterization of disease severity and phenotype.

Cardiopulmonary exercise testing (CPET) remains the reference tool for the evaluation of functional capacity and prognostic stratification in chronic HF, with robust clinical evidence supporting its use in both HFrEF and HFpEF. By providing an integrated assessment of cardiovascular, ventilatory, and metabolic responses to exercise, CPET helps identify the principal mechanisms limiting performance and offers important information for both clinical management and therapeutic monitoring. In patients with chronic HF, several CPET-derived variables have consistently been associated with worse functional status and adverse prognosis, particularly in the HFrEF population, where they guide heart transplant and VAD eligibility. These variables include reduced peak oxygen uptake (VO_2_peak), an abnormally flat oxygen pulse response, an increased heart rate/VO_2_ relationship, oscillatory exercise ventilation, an early occurrence of the second ventilatory threshold, an elevated VE/VCO_2_ slope, and a blunted blood pressure response during exercise. Taken together, these abnormalities reflect impaired central hemodynamic reserve, inefficient ventilatory adaptation, and reduced overall exercise efficiency [14,15]. Stress echocardiography provides complementary mechanistic information by directly assessing cardiac structure and hemodynamic behavior during exertion. In HF with preserved ejection fraction (HFpEF), where resting hemodynamics are often inconclusive, exercise echocardiography is the clinical cornerstone for diagnosis. In this setting, an exercise-induced increase in E/e′ ratio and systolic pulmonary artery pressure is frequently observed and may support the identification of elevated left ventricular filling pressures and impaired diastolic reserve. In contrast, in HF with reduced ejection fraction (HFrEF), stress echocardiography more commonly highlights impaired contractile reserve, limited stroke volume augmentation, right ventricular dysfunction, and abnormal pulmonary hemodynamic responses. Parameters such as TAPSE, left ventricular ejection fraction, myocardial strain, and pulmonary pressures may therefore provide important insights into disease severity and exercise intolerance [7,16]. NIRS adds a further layer of pathophysiological interpretation by exploring the peripheral component of exercise limitation. In both chronic and acute HF, skeletal muscle oxygenation may be impaired because of reduced perfusion, endothelial dysfunction, abnormal microvascular regulation, and altered oxidative metabolism. NIRS may detect these abnormalities even when overt peripheral hypoperfusion is not clinically evident, thus providing clinically relevant information on regional oxygen delivery and extraction. This may be particularly useful in identifying patients in whom peripheral factors substantially contribute to reduced exercise capacity, as well as in monitoring response to interventions aimed at improving muscle perfusion or oxidative function. The combined use of CPET, stress echocardiography, and NIRS therefore allows a multidimensional characterization of exercise intolerance in HF [17,18]. By integrating systemic performance, central hemodynamics, and peripheral oxygen handling, this approach may improve phenotypic discrimination across HFrEF, HFpEF, and mixed or predominantly peripheral forms of limitation. Such an integrated framework may also facilitate a more individualized therapeutic strategy, supporting targeted interventions directed toward central hemodynamics, pulmonary pressures, or peripheral conditioning, according to the dominant mechanism of impairment (Table 1). This may be particularly relevant when tailoring exercise rehabilitation programs, optimizing decongestive or hemodynamic therapies, and monitoring functional response over time.

### 5.2. Pulmonary Hypertension

Pulmonary hypertension (PH) is a hemodynamic disorder characterized by abnormally elevated pulmonary arterial pressure and increased right ventricular (RV) afterload. It encompasses a heterogeneous group of conditions arising from primary pulmonary vascular disease, left heart disease, chronic lung disorders, thromboembolic disease, or systemic disorders. Regardless of etiology, exercise intolerance is a cardinal feature of PH and reflects the complex interaction among impaired pulmonary vascular reserve, limited right ventricular adaptation, ventilatory inefficiency, and peripheral abnormalities in oxygen delivery and utilization. In this setting, an integrated functional approach may provide a more comprehensive characterization of disease severity and pathophysiological phenotype.

CPET plays a central role in the evaluation of functional limitation in PH because it allows simultaneous assessment of cardiovascular, respiratory, and metabolic responses to exercise [15].

Reduced peak oxygen uptake is a hallmark finding and reflects the inability to adequately augment cardiac output during exertion as a consequence of increased pulmonary vascular resistance and impaired RV-pulmonary arterial coupling.

In parallel, an increased VE/VCO_2_ slope is commonly observed and indicates ventilatory inefficiency, largely related to abnormal ventilatory-perfusion matching, increased physiological dead space, and enhanced chemoreflex drive.

Additional abnormalities may include reduced end-tidal carbon dioxide, early anaerobic transition, and impaired oxygen pulse behavior, all of which contribute to the recognition of a hemodynamic limitation to exercise. In PH, the inability of the right ventricle to appropriately increase forward flow during effort may result in reduced skeletal muscle perfusion and an early imbalance between oxygen delivery and utilization. Accordingly, patients with PH may exhibit a rapid and pronounced decline in tissue saturation index (TSI) even at low workloads, delayed post-exercise recovery of tissue oxygenation, and abnormal oxyhemoglobin and deoxyhemoglobin kinetics consistent with increased peripheral oxygen extraction in the setting of inadequate convective oxygen transport. These findings may help identify the peripheral consequences of RV dysfunction and further characterize the mechanisms contributing to exercise intolerance [19].

Exercise echocardiography provides the central hemodynamic counterpart to CPET and NIRS by allowing direct evaluation of right ventricular performance and pulmonary pressure response during physical stress. In patients with PH, exercise imaging may reveal an exaggerated rise in systolic pulmonary artery pressure, increased tricuspid regurgitation velocity, impaired RV contractile reserve, and abnormal changes in right ventricular outflow tract velocity-time integral, reflecting limited forward flow adaptation. In addition, the dynamic assessment of RV size, function, and RV-pulmonary arterial coupling may offer valuable insight into the severity of hemodynamic compromise during exertion [20] (Table 2).

### 5.3. Peripheral Artery Disease

Peripheral artery disease (PAD) is a clinical manifestation of systemic atherosclerosis characterized by obstructive lesions of the lower-extremity arteries, leading to impaired limb perfusion during exercise and, in more advanced stages, ischemia at rest. Although the ankle–brachial index (ABI) remains the cornerstone of initial diagnosis, functional assessment in PAD increasingly requires complementary tools capable of characterizing the physiological consequences of impaired arterial flow during exertion. In this context, cardiopulmonary exercise testing (CPET), near-infrared spectroscopy (NIRS), and stress echocardiography may provide synergistic information on global exercise performance, peripheral muscle ischemia, and the potential contribution of concomitant cardiac dysfunction. Although walking limitation in PAD is primarily related to impaired limb perfusion and skeletal muscle ischemia, exercise intolerance often reflects the combined effect of peripheral vascular obstruction, deconditioning, endothelial dysfunction, and coexisting cardiovascular disease. CPET therefore helps quantify the overall reduction in functional capacity, identify the relative contribution of circulatory and ventilatory limitation, and monitor the effects of exercise rehabilitation or revascularization [21]. In addition, CPET may be particularly informative in patients with atypical symptoms or multiple comorbidities, in whom the distinction between central and peripheral determinants of reduced performance may not be clinically evident. In patients with PAD, Stress echocardiography is particularly useful for detecting concomitant myocardial ischemia, assessing left ventricular systolic and diastolic reserve, and identifying whether exercise intolerance is partly related to a cardiac rather than exclusively peripheral limitation. Given the high prevalence of coronary artery disease and shared atherosclerotic burden in this population, stress echocardiography may substantially refine the interpretation of exertional symptoms and support a more comprehensive cardiovascular evaluation. In PAD, reduced arterial perfusion limits oxygen delivery to active muscle, leading to an accelerated decline in tissue saturation during exercise and delayed restoration of oxygenation after effort cessation. These dynamic abnormalities may be detected even in subjects with resting ABI values within the normal range, thereby supporting earlier identification of functionally significant disease [22,23]. Furthermore, NIRS may be useful for quantifying the severity of exercise-induced ischemia and for monitoring response to supervised exercise therapy or revascularization procedures (Table 3).

### 5.4. Sports Cardiology and Athletic Performance Assessment

Exercise testing plays a central role in sports cardiology, where it serves both diagnostic and performance-oriented purposes. Beyond the detection of previously unrecognized cardiovascular abnormalities, exercise-based assessment is essential for the evaluation of physiological adaptation to training, characterization of athletic performance, and optimization of individualized exercise prescription. In trained athletes, chronic exposure to exercise induces a series of cardiovascular adaptations collectively referred to as the “athlete’s heart,” including increased left ventricular end-diastolic volume, eccentric remodeling, resting bradycardia, and augmented maximal cardiac output. Distinguishing these physiological adaptations from pathological conditions, particularly cardiomyopathies and exercise-induced arrhythmogenic substrates, is a key challenge in sports cardiology, and multimodal stress evaluation may substantially improve diagnostic accuracy [24].

In athletes, CPET provides objective quantification of aerobic capacity, ventilatory efficiency, and exercise thresholds, thereby allowing detailed assessment of performance profile, training adaptation, and potential physiological limitations. It is also useful for monitoring the effects of training over time and for identifying abnormal responses suggestive of occult cardiovascular or pulmonary disease.

In the differential diagnosis between physiological athletic remodeling and cardiomyopathy, CPET may provide particularly valuable supportive information. In athletes with left ventricular hypertrophy or equivocal structural findings, a peak VO_2_ greater than 50 mL/kg/min or greater than 120% of the predicted value may support the interpretation of physiological athletic remodeling rather than hypertrophic cardiomyopathy in selected contexts. However, this parameter should never be considered in isolation and must be interpreted within a comprehensive multimodal framework including sex, age, sport discipline, body size, ethnicity, training history, symptoms, family history, electrocardiographic findings, imaging phenotype, and genetic background [25].

In athletes, exercise is typically associated with a progressive reduction in tissue saturation index (TSI), reflecting increased muscular oxygen extraction. The pattern and magnitude of this response, as well as post-exercise recovery kinetics, may provide insight into peripheral vascular function, muscle oxidative capacity, and training status. In addition, the identification of a hemoglobin difference breakpoint may help characterize metabolic transition points and complement conventional threshold analysis derived from CPET. In the athletic population, stress echocardiography is particularly valuable when there is a need to distinguish physiological remodeling from cardiomyopathic phenotypes, such as hypertrophic cardiomyopathy, or to investigate exertional symptoms that are not explained by resting studies alone [2,26].

It may also detect exercise-induced myocardial ischemia or abnormal contractile responses even in the absence of overt electrocardiographic changes, thereby contributing to the identification of latent cardiovascular disease in selected athletes (Table 4).

### 5.5. Cardiomyopathies

CPET remains the reference method for the objective evaluation of functional capacity in cardiomyopathies, providing a global measure of disease severity and an important framework for prognostic assessment. In hypertrophic cardiomyopathy (HCM), exercise limitation is frequently multifactorial and may reflect impaired diastolic reserve, dynamic left ventricular outflow tract obstruction, chronotropic incompetence, abnormal ventilatory efficiency, or impaired peripheral oxygen utilization. Reduced peak VO_2_ and an increased VE/VCO_2_ slope have consistently been associated with worse functional status and adverse outcomes.

In dilated cardiomyopathy, CPET-derived variables, particularly percent-predicted peak VO_2_ and ventilatory efficiency indices, provide relevant prognostic information and may contribute to the timing of advanced heart failure therapies, including referral for transplant evaluation.

NIRS adds a peripheral dimension to this evaluation, identifying abnormalities in local oxygen delivery, extraction, and reoxygenation kinetics that are not fully captured by central cardiopulmonary indices alone [27,28]. Such information may be particularly relevant when peripheral maladaptation, microvascular dysfunction, or impaired oxidative metabolism contribute substantially to exercise intolerance. NIRS-derived changes in tissue saturation index during exercise may correlate with exercise capacity and may help identify microcirculatory impairment beyond the degree of central cardiac dysfunction. In addition, NIRS-derived breakpoints have been proposed as complementary markers of metabolic transition, showing potential agreement with threshold determination based on ventilatory methods and offering a further tool for individualized exercise prescription [29].

Stress echocardiography completes the multimodal assessment by directly interrogating cardiac mechanics and hemodynamic behavior during stress. When integrated with CPET, this approach permits simultaneous evaluation of gas exchange and dynamic cardiac function, including systolic and diastolic reserve, stroke volume adaptation, pulmonary pressure response, exercise-induced valvular abnormalities, and, when relevant, pulmonary congestion. In HCM, stress echocardiography is particularly valuable for identifying dynamic left ventricular outflow tract obstruction, abnormal filling pressure response, and exercise-induced pulmonary hypertension, all of which may contribute substantially to symptoms and reduced functional capacity. In dilated cardiomyopathy, the demonstration of preserved contractile reserve has been associated with a more favorable clinical course, whereas impaired augmentation of systolic performance during exercise may identify patients at higher risk of progression and adverse events [29].

Recent work from our group has further highlighted the clinical value of this integrated strategy in both Fabry disease and HCM. In Fabry cardiomyopathy, combined CPET and stress echocardiography, with adjunctive NIRS assessment in selected protocols, has shown that exercise intolerance may result from a heterogeneous interplay between cardiac, pulmonary-vascular, and peripheral mechanisms rather than from myocardial involvement alone. These studies have helped reveal distinct functional phenotypes, including sex-related differences in the relative contribution of central versus peripheral limitation, and have supported the development of a more mechanism-oriented classification of exercise impairment [30].

Similarly, in HCM, our combined CPET-stress echocardiographic studies have shown that reduced aerobic capacity is closely linked to impaired diastolic reserve, abnormal pulmonary pressure response, and adverse hemodynamic adaptation during exercise, thereby improving phenotypic characterization beyond resting imaging alone and reinforcing the prognostic value of an integrated stress-based evaluation [31] (Table 5).

### 5.6. Practical Considerations and Barriers to Clinical Implementation

While the multimodal integration of CPET, ESE, and NIRS offers a comprehensive view of the oxygen transport chain, several methodological constraints currently limit its widespread adoption. The operational costs and equipment burden are significantly higher than standard testing, and the technical complexity introduces major challenges, such as the need for precise temporal synchronization between devices to ensure accurate data alignment. Furthermore, echocardiographic image quality often degrades during peak exertion due to hyperventilation and chest wall movement, frequently requiring a compromise in maximal workload to facilitate diagnostic imaging. This necessitates a careful choice of exercise modality; for instance, the trade-offs between upright and semi-supine ergometry must be considered, as the latter improves acoustic windows but significantly alters central hemodynamics and preload compared to standard upright protocols. Beyond hardware limitations, the interpretation of NIRS data is currently hampered by significant inter-device variability and a lack of strong evidence across different age and disease populations. The successful implementation of this “triple” assessment also requires specialized personnel, intensive training, and a steep learning curve to manage the integrated interpretation of potentially discordant findings. Patient safety remains a priority, demanding strict adherence to contraindications and a robust clinical infrastructure, particularly in advanced heart failure and cardiomyopathy cohorts. Nevertheless, while routine implementation may currently face barriers in general practice, this multimodal protocol could represent the new gold standard for tertiary centers dedicated to the comprehensive management of complex cardiomyopathies, sports cardiology, athletic performance assessment and advanced heart failure.

## 6. Conclusions

The integration of CPET, stress echocardiography, and NIRS represents an evolving approach to the assessment of exercise intolerance by combining systemic functional evaluation, central hemodynamic assessment, and peripheral muscle oxygenation analysis. Compared with conventional CPET alone, this multimodal strategy may provide a more comprehensive interpretation of exercise limitation and improve the characterization of the mechanisms underlying impaired functional capacity.

Potential applications span several clinical settings, including heart failure, pulmonary hypertension, peripheral artery disease, cardiomyopathies, and sports cardiology. However, important limitations remain, including the lack of standardized protocols, technical complexity, operator dependency, and the limited availability of integrated platforms. In addition, although several physiological and clinical observations are promising, the incremental diagnostic, prognostic, and therapeutic value of combined CPET–stress echocardiography–NIRS assessment still requires validation in larger prospective studies.

Future research should therefore focus on protocol harmonization, validation of multimodal parameters, and definition of the most clinically meaningful applications of this integrated approach.

## Figures and Tables

**Table 1:** Key findings from integrated CPET, stress echocardiography and NIRS assessment in heart failure.

Technique/Parameter	Typical Finding in Heart Failure	Clinical Significance
CPET: Peak VO_2_	Reduced	Reflects impaired global aerobic capacity and remains a major prognostic marker in HF.
CPET: VE/VCO_2_ slope	Increased	Indicates ventilatory inefficiency and is associated with greater disease severity and worse outcomes.
CPET: Oxygen pulse	Reduced and/or flattened during exercise	Suggests impaired stroke volume augmentation and/or reduced peripheral oxygen extraction.
CPET: Exercise oscillatory ventilation	May be present	Marker of advanced cardiocirculatory dysfunction and unfavorable prognosis.
CPET: Chronotropic and blood pressure reserve	Abnormal	Suggests reduced cardiovascular reserve and more severe hemodynamic limitation.
Stress echocardiography: Contractile reserve	Reduced	Supports impaired systolic reserve, more commonly observed in HFrEF.
Stress echocardiography: Filling pressures (E/e′)	Increased during exercise	Suggests impaired diastolic reserve and elevated LV filling pressures, particularly relevant in HFpEF.
Stress echocardiography: Pulmonary pressures (sPAP/PASP)	Increased during exercise	Indicates abnormal pulmonary hemodynamic response and/or elevated left-sided filling pressures contributing to exertional dyspnea.
Stress echocardiography: Functional valvular regurgitation	May worsen with exercise	Reflects dynamic loading abnormalities and may contribute to reduced exercise tolerance.
NIRS: Resting TSI	Normal or mildly reduced	Resting values may be less informative than exercise and recovery kinetics.
NIRS: TSI decline during exercise	Faster and/or more pronounced	Suggests impaired matching between local oxygen delivery and utilization.
NIRS: Post-exercise recovery	Delayed	Consistent with impaired restoration of muscle oxygen homeostasis and altered oxidative function.
NIRS: tHb response	Blunted increase or minimal variation	Suggests limited local blood-volume recruitment and reduced muscle perfusion during exercise.
NIRS: HHb kinetics/DiffHb breakpoint	Abnormal pattern	Reflects altered peripheral O_2_ extraction; breakpoint may parallel late metabolic transition, although standardization remains limited.

**Abbreviations:** CPET, cardiopulmonary exercise testing; DiffHb, difference between oxygenated and deoxygenated hemoglobin; HF, heart failure; HFpEF, heart failure with preserved ejection fraction; HFrEF, heart failure with reduced ejection fraction; HHb, deoxygenated hemoglobin; LV, left ventricular; PASP/sPAP, pulmonary artery systolic pressure; TSI, tissue saturation index; VE/VCO_2_ slope, relationship between minute ventilation and carbon dioxide production; VO_2_, oxygen uptake.

**Table 2:** Integrated CPET and stress echocardiography findings in pulmonary hypertension.

Technique	Parameter	Typical Finding in PH	Clinical Significance
**CPET**	Peak VO_2_	Reduced	Reflects impaired aerobic capacity and reduced ability to augment cardiac output during exercise because of increased pulmonary vascular load and limited RV–pulmonary arterial coupling.
**CPET**	VE/VCO_2_ slope	Increased	Indicates ventilatory inefficiency related to increased dead space, ventilation–perfusion mismatch, and enhanced ventilatory drive.
**CPET**	PetCO_2_ at rest and/or during exercise	Reduced	Suggests impaired pulmonary perfusion and inefficient CO_2_ delivery to the alveoli, typically reflecting ventilation–perfusion abnormalities and increased dead space.
**CPET**	O_2_ pulse (VO_2_/HR)	Reduced	Indirectly reflects impaired stroke volume augmentation and reduced forward flow during exercise.
**CPET**	VE/VO_2_	Increased	Consistent with an excessive ventilatory requirement for a given oxygen uptake and inefficient ventilatory adaptation during effort.
**CPET**	Anaerobic threshold	Early, reduced, or not clearly identifiable	Reflects reduced cardiopulmonary reserve and premature transition to anaerobic metabolism.
**CPET**	Exercise SpO_2_	May decrease during exertion	Supports worsening gas exchange and ventilation–perfusion mismatch during exercise.
**Stress echocardiography**	Systolic pulmonary artery pressure (sPAP/PASP)	Increased at rest and/or exaggerated rise during exercise	Reflects abnormal pulmonary vascular load and impaired adaptation of the pulmonary circulation to effort.
**Stress echocardiography**	Tricuspid regurgitation velocity (TRV)	Increased	Main Doppler surrogate for pulmonary pressure estimation and marker of elevated RV systolic pressure within the overall echocardiographic context.
**Stress echocardiography**	Right ventricular contractile reserve	Reduced	Suggests impaired ability of the right ventricle to augment systolic function during exercise.
**Stress echocardiography**	RVOT velocity–time integral (RVOT VTI)	Reduced or blunted increase during exercise	Indicates limited forward flow augmentation across the RV outflow tract and may reflect impaired stroke volume reserve.
**Stress echocardiography**	TRV/RVOT VTI ratio	Increased	May suggest increased pulmonary vascular resistance and an unfavorable relation between pressure generation and forward flow.
**Stress echocardiography**	TAPSE or RV longitudinal function	Reduced augmentation during exercise	Reflects impaired longitudinal RV reserve and reduced systolic adaptation to increased afterload.
**Stress echocardiography**	RV–pulmonary arterial coupling	Impaired	Identifies inefficient hemodynamic adaptation to exercise and may have prognostic relevance.

**Abbreviations:** CPET, cardiopulmonary exercise testing; PASP/sPAP, pulmonary artery systolic pressure; PetCO_2_, end-tidal carbon dioxide pressure; PH, pulmonary hypertension; RV, right ventricular; RVOT VTI, right ventricular outflow tract velocity–time integral; SpO_2_, peripheral oxygen saturation; TAPSE, tricuspid annular plane systolic excursion; TRV, tricuspid regurgitation velocity; VE/VCO_2_ slope, relationship between minute ventilation and carbon dioxide production; VE/VO_2_, relationship between minute ventilation and oxygen uptake; VO_2_, oxygen uptake.

**Table 3:** Main findings derived from integrated CPET, NIRS and stress echocardiography in peripheral artery disease.

Modality	Key Parameter	Typical Finding in PAD	Pathophysiological/Clinical Significance
**CPET**	Peak VO_2_	Reduced	Reflects impaired global exercise capacity, resulting from the combined effects of limb ischemia, deconditioning, endothelial dysfunction, and frequent cardiovascular comorbidities.
**CPET**	Anaerobic threshold (AT)	Early onset/reduced	Suggests reduced functional reserve and earlier transition to anaerobic metabolism during exercise.
**CPET**	Oxygen pulse	Reduced	May indicate limited stroke volume augmentation and/or reduced peripheral oxygen utilization during exercise; interpretation should consider concomitant cardiac disease.
**CPET**	VE/VCO_2_ slope	Normal or mildly increased; may be higher in selected patients	Usually less specific for PAD itself, but may suggest inefficient ventilatory adaptation or associated cardiopulmonary comorbidity.
**NIRS**	ΔTSI during exercise	More rapid and pronounced decline	Marker of exercise-induced muscle ischemia and impaired local oxygen delivery to the active limb musculature.
**NIRS**	TSI recovery time	Prolonged	Reflects delayed reoxygenation after exercise and is consistent with impaired vascular reserve and microvascular function.
**NIRS**	HHb response	Increased	Suggests enhanced local oxygen extraction in the setting of reduced convective oxygen delivery.
**Stress echocardiography**	Ventricular reserve/wall motion	Usually preserved unless concomitant cardiac disease is present	Useful to exclude inducible myocardial ischemia or central cardiac limitation as contributors to exercise intolerance in PAD.

**Abbreviations:** AT, anaerobic threshold; HHb, deoxygenated hemoglobin; NIRS, near-infrared spectroscopy; PAD, peripheral artery disease; TSI, tissue saturation index; VE/VCO_2_ slope, relationship between minute ventilation and carbon dioxide production; VO_2_, oxygen uptake.

**Table 4:** Key findings from integrated CPET, stress echocardiography and NIRS assessment in sports cardiology and athletic performance assessment.

Modality	Key Parameter	Typical Finding in Trained Athletes/Abnormal Pattern of Interest	Pathophysiological/Clinical Significance
CPET	Peak VO2	Usually markedly elevated in trained athletes; values > 50 mL/kg/min or >120% of predicted may support physiological athletic remodeling rather than hypertrophic cardiomyopathy in athletes with equivocal left ventricular hypertrophy	Reflects high global aerobic capacity and may aid in differentiating athlete’s heart from cardiomyopathic phenotypes when interpreted within a multimodality framework.
CPET	Ventilatory thresholds (VT1/VT2)	Delayed occurrence at higher workloads	Indicates superior aerobic efficiency and training adaptation; abnormal early thresholds may suggest deconditioning or occult disease.
CPET	VE/VCO2 slope	Typically normal or low–normal in healthy athletes	Preserved ventilatory efficiency; abnormal elevation should raise suspicion for cardiopulmonary limitation or inefficient exercise response.
CPET	Oxygen pulse	Increased with progressive exercise	Surrogate of effective stroke volume augmentation and peripheral oxygen extraction during exertion; a flattened pattern may suggest cardiovascular limitation.
NIRS	TSI decline during exercise	Progressive fall during increasing workload	Reflects physiological augmentation of skeletal muscle oxygen extraction; the pattern may help characterize local metabolic stress and muscular efficiency.
NIRS	Recovery kinetics/reoxygenation time	Rapid post-exercise recovery in well-conditioned athletes	Provides information on peripheral vascular responsiveness and muscle oxidative capacity; delayed recovery may suggest impaired peripheral conditioning or incomplete recovery status.
NIRS	HHb/DiffHb breakpoint	Detectable near metabolic transition zones	May complement CPET threshold analysis by identifying peripheral metabolic transition points during incremental exercise.
Stress echocardiography	LV systolic and diastolic reserve	Preserved or enhanced augmentation during exercise	Supports physiological adaptation to training; impaired reserve may suggest underlying myocardial disease.
Stress echocardiography	Regional wall motion	Normal in physiological adaptation	Useful for detecting exercise-induced ischemia or occult structural heart disease in symptomatic or high-risk athletes.
Stress echocardiography	Dynamic LVOT gradient/valvular behavior	Usually absent or physiologically mild	Helps distinguish athlete’s heart from hypertrophic cardiomyopathy or other structural causes of exertional symptoms.
Integrated interpretation	CPET + ESE + NIRS profile	Concordant high performance with preserved central reserve and efficient peripheral oxygen handling	Supports physiological athletic adaptation; discrepant central or peripheral findings may orient toward occult pathology, maladaptation, or suboptimal training response.

Abbreviations: CPET, cardiopulmonary exercise testing; DiffHb, difference between oxygenated and deoxygenated hemoglobin; ESE, exercise stress echocardiography; HHb, deoxygenated hemoglobin; LV, left ventricular; LVOT, left ventricular outflow tract; NIRS, near-infrared spectroscopy; TSI, tissue saturation index; VE/VCO_2_ slope, relationship between minute ventilation and carbon dioxide production; VO_2_, oxygen uptake; VT1/VT2, first and second ventilatory thresholds.

**Table 5:** Main findings derived from integrated CPET, stress echocardiography and NIRS in cardiomyopathies.

Modality	Key Parameter	Typical Finding in Cardiomyopathies	Pathophysiological/Clinical Significance
**CPET**	Peak VO_2_	Reduced	Reflects impaired global exercise capacity and is a major marker of functional limitation and prognosis across cardiomyopathy phenotypes.
**CPET**	Percent-predicted peak VO_2_	Reduced	Particularly useful for risk stratification, especially in dilated cardiomyopathy, where it may support timing of advanced heart failure therapies.
**CPET**	VE/VCO_2_ slope	Increased	Indicates ventilatory inefficiency and is associated with worse functional status and adverse outcomes.
**CPET**	Chronotropic response	Abnormal or blunted	Suggests impaired cardiovascular adaptation to exercise and may contribute to exercise intolerance.
**CPET**	Ventilatory thresholds	Early or reduced	Reflect reduced cardiopulmonary reserve and premature metabolic transition during effort.
**NIRS**	TSI decline during exercise	Faster and/or more pronounced	Suggests impaired balance between peripheral oxygen delivery and utilization, possibly related to microvascular dysfunction or altered oxidative metabolism.
**NIRS**	Recovery kinetics	Delayed	Indicates impaired restoration of muscle oxygen homeostasis and may reflect reduced oxidative capacity.
**NIRS**	HHb/DiffHb breakpoint	Detectable or abnormal	May complement ventilatory threshold assessment and provide insight into peripheral metabolic transition.
**NIRS**	Peripheral oxygenation profile	Abnormal despite modest central limitation in some patients	Supports the contribution of skeletal muscle and microcirculatory dysfunction to exercise intolerance.
**Stress echocardiography**	Contractile reserve	Reduced in advanced disease; preserved reserve associated with better outcome	Helps stratify systolic functional reserve and prognosis, particularly in dilated cardiomyopathy.
**Stress echocardiography**	Diastolic reserve/filling pressures	Abnormal increase during exercise	Indicates impaired diastolic adaptation and contributes to exertional dyspnea and reduced exercise tolerance.
**Stress echocardiography**	LVOT obstruction	Dynamic increase during exercise in selected patients	Particularly relevant in hypertrophic cardiomyopathy, where exercise-induced obstruction may be a major determinant of symptoms.
**Stress echocardiography**	Pulmonary pressure response	Increased during exercise	Suggests abnormal hemodynamic adaptation and may identify patients with more advanced functional limitation.
**Stress echocardiography**	Exercise B-lines/pulmonary congestion	May appear or increase with stress	Supports elevated filling pressures and exercise-induced pulmonary congestion.
**Integrated interpretation**	CPET + ESE + NIRS profile	Concordant central and/or peripheral abnormalities	Improves phenotypic characterization, refines risk stratification, and supports individualized therapeutic and exercise prescription strategies.

**Abbreviations:** CPET, cardiopulmonary exercise testing; DiffHb, difference between oxygenated and deoxygenated hemoglobin; ESE, exercise stress echocardiography; HHb, deoxygenated hemoglobin; LVOT, left ventricular outflow tract; NIRS, near-infrared spectroscopy; TSI, tissue saturation index; VE/VCO_2_ slope, relationship between minute ventilation and carbon dioxide production; VO_2_, oxygen uptake.

## Data Availability

No new data were created or analyzed in this narrative review.

## References

[1] Guazzi M., Arena R., Halle M., Piepoli M.F., Myers J., Lavie C.J. (2018). 2016 focused update: Clinical recommendations for cardiopulmonary exercise testing data assessment in specific patient populations. Eur. Heart J..

[2] Malhotra R., Bakken K., D’Elia E., Lewis G.D. (2016). Cardiopulmonary Exercise Testing in Heart Failure. JACC Heart Fail..

[3] Ferrari M., Mottola L., Quaresima V. (2004). Principles, techniques, and limitations of near-infrared spectroscopy. Can. J. Appl. Physiol..

[4] Hamaoka T., McCully K.K., Quaresima V., Yamamoto K., Chance B. (2007). Near-infrared spectroscopy/imaging for monitoring muscle oxygenation and oxidative metabolism in healthy and diseased humans. J. Biomed. Opt..

[5] Agostoni P., Dumitrescu D. (2019). How to perform and report a cardiopulmonary exercise test in patients with chronic heart failure. Int. J. Cardiol..

[6] Salvioni E., Bonomi A., Magrì D., Merlo M., Pezzuto B., Chiesa M., Mapelli M., Baracchini N., Sinagra G., Piepoli M. (2023). The cardiopulmonary exercise test in the prognostic evaluation of patients with heart failure and cardiomyopathies: The long history of making a one-size-fits-all suit. Eur. J. Prev. Cardiol..

[7] Lancellotti P., Pellikka P.A., Budts W., Chaudhry F.A., Donal E., Dulgheru R., Edvardsen T., Garbi M., Ha J.W., Kane G.C. (2016). The clinical use of stress echocardiography in non-ischaemic heart disease: Recommendations from the European Association of Cardiovascular Imaging and the American Society of Echocardiography. Eur. Heart J. Cardiovasc. Imaging.

[8] Lang R.M., Badano L.P., Mor-Avi V., Afilalo J., Armstrong A., Ernande L., Flachskampf F.A., Foster E., Goldstein S.A., Kuznetsova T. (2015). Recommendations for cardiac chamber quantification by echocardiography in adults: An update from the American Society of Echocardiography and the European Association of Cardiovascular Imaging. Eur. Heart J. Cardiovasc. Imaging.

[9] Adami A., Rossiter H.B. (2018). Principles, insights, and potential pitfalls of the noninvasive determination of muscle oxidative capacity by near-infrared spectroscopy. J. Appl. Physiol..

[10] Tandirerung F.J., Jamieson A., Hendrick E., Hughes A.D., Jones S. (2024). Near-infrared spectroscopy (NIRS) in vivo assessment of skeletal muscle oxidative capacity: A comparison of results from short versus long exercise protocols and reproducibility in non-athletic adults. Front. Physiol..

[11] Dorobantu D.M., Wadey C.A., Berryman B., Amir N.H., Forsythe L., Stuart A.G., Pieles G.E., Williams C.A. (2024). A new protocol for a single-stage combined cardiopulmonary and echocardiography exercise test: A pilot study. Eur. Heart J. Imaging Methods Pract..

[12] Smarz K., Jaxa-Chamiec T., Zaborska B., Tysarowski M., Budaj A. (2021). Mechanisms of Exercise Capacity Improvement after Cardiac Rehabilitation Following Myocardial Infarction Assessed with Combined Stress Echocardiography and Cardiopulmonary Exercise Testing. J. Clin. Med..

[13] Del Punta L., De Biase N., Armenia S., Di Fiore V., Maremmani D., Gargani L., Mazzola M., De Carlo M., Mengozzi A., Lomonaco T. (2023). Combining cardiopulmonary exercise testing with echocardiography: A multiparametric approach to the cardiovascular and cardiopulmonary systems. Eur. Heart J. Imaging Methods Pract..

[14] Karsten M., Salvioni E., Palermo P., Mattavelli I., Scatigna M., Mapelli M., Grilli G., Pezzuto B., Apostolo A., Ribeiro G.D.S. (2025). Periodic breathing during exercise in heart failure: Beyond the classic risk factors. Eur. Heart J. Suppl..

[15] Balady G.J., Arena R., Sietsema K., Myers J., Coke L., Fletcher G.F., Forman D., Franklin B., Guazzi M., Gulati M. (2010). Clinician’s Guide to cardiopulmonary exercise testing in adults: A scientific statement from the American Heart Association. Circulation.

[16] Pálinkás E.D., Re F., Peteiro J., Tesic M., Pálinkás A., Torres M.A.R., Dikic A.D., Beleslin B., Van De Heyning C.M., D’Alfonso M.G. (2022). Pulmonary congestion during Exercise stress Echocardiography in Hypertrophic Cardiomyopathy. Int. J. Cardiovasc. Imaging.

[17] Hanada A., Okita K., Yonezawa K., Ohtsubo M., Kohya T., Murakami T., Nishijima H., Tamura M., Kitabatake A. (2000). Dissociation between muscle metabolism and oxygen kinetics during recovery from exercise in patients with chronic heart failure. Heart.

[18] Reddy Y.N.V., Carter R.E., Obokata M., Redfield M.M., Borlaug B.A. (2018). A Simple, Evidence-Based Approach to Help Guide Diagnosis of Heart Failure with Preserved Ejection Fraction. Circulation.

[19] Biondi F., Fiore V.D., Pancani R., Carrozzi L., De Caterina R., Madonna R. (2026). Exercise testing in chronic thromboembolic pulmonary hypertension and chronic thromboembolic pulmonary disease without pulmonary hypertension: A comprehensive systematic review and meta-analysis. Eur. J. Intern. Med..

[20] Gargani L., Pugliese N.R., De Biase N., Mazzola M., Agoston G., Arcopinto M., Argiento P., Armstrong W.F., Bandera F., Cademartiri F. (2023). Exercise Stress Echocardiography of the Right Ventricle and Pulmonary Circulation. J. Am. Coll. Cardiol..

[21] Koirala B., Concas A., Cincotti A., Sun Y., Hernández A., Goodwin M.L., Gladden L.B., Lai N. (2024). Estimation of differential pathlength factor from NIRS measurement in skeletal muscle. Respir. Physiol. Neurobiol..

[22] Pilotto A.M., Adami A., Mazzolari R., Brocca L., Crea E., Zuccarelli L., Pellegrino M.A., Bottinelli R., Grassi B., Rossiter H.B. (2022). Near-infrared spectroscopy estimation of combined skeletal muscle oxidative capacity and O_2_ diffusion capacity in humans. J. Physiol..

[23] Link M.S., Estes N.A.M., Maron B.J. (2015). Eligibility and Disqualification Recommendations for Competitive Athletes with Cardiovascular Abnormalities: Task Force 13: Commotio Cordis: A Scientific Statement from the American Heart Association and American College of Cardiology. J. Am. Coll. Cardiol..

[24] Piepoli M.F., Guazzi M., Boriani G., Cicoira M., Corrà U., Dalla Libera L., Emdin M., Mele D., Passino C., Vescovo G. (2010). Exercise intolerance in chronic heart failure: Mechanisms and therapies. Part I Eur. J. Cardiovasc. Prev. Rehabil..

[25] Mapelli M., Salvioni E., Mattavelli I., Vignati C., Galotta A., Magrì D., Apostolo A., Sciomer S., Campodonico J., Agostoni P. (2023). Cardiopulmonary exercise testing and heart failure: A tale born from oxygen uptake. Eur. Heart J. Suppl..

[26] Wagner P.D. (2015). The physiological basis of pulmonary gas exchange: Implications for clinical interpretation of arterial blood gases. Eur. Respir. J..

[27] Willixhofer R., Mapelli M., Baracchini N., Campana N., Capovilla T.M., Nava A., Salvioni E., Vignati C., Rubbo F.M., Magrì D. (2025). Cardiopulmonary exercise testing in hypertrophic cardiomyopathy: The role of reduced O_2_ pulse and chronotropic incompetence in myocardial adaptation. Eur. J. Prev. Cardiol..

[28] Willixhofer R., Contini M., Emdin M., Magrì D., Bonomi A., Salvioni E., Celeste F., Del Torto A., Passino C., Capelle C.D.J. (2025). Exercise limitations in amyloid cardiomyopathy assessed by cardiopulmonary exercise testing-A multicentre study. ESC Heart Fail..

[29] Su Y., Li C., Peng Q., Yin L. (2025). Exercise Stress Echocardiography Predicts Adverse Cardiovascular Events in Hypertrophic Cardiomyopathy: A 5-Year Prospective Study. Rev. Cardiovasc. Med..

[30] Mistrulli R., Re F., Giacalone G., di Francesco M., Ferri G., Beato S., Onorato F., Lanzillo C., Canali E., Piepoli M. (2025). Cardiopulmonary exercise test and stress echocardiography in Fabry cardiomyopathy: Insights into functional impairment and risk stratification. Eur. Heart J. Qual. Care Clin. Outcomes.

[31] Mistrulli R., Re F., Giacalone G., Storozhenko T., Ciacci P., Di Cristo A., Ferrera A., Gabrielli D., Halasz G. (2026). Severe LVOT obstruction in HCM: Effects on exercise capacity and outcomes from cardiopulmonary exercise testing combined with stress echocardiography. Eur. Heart J. Qual. Care Clin. Outcomes.

